# Mapping standard ophthalmic outcome sets to metrics currently reported in eight eye hospitals

**DOI:** 10.1186/s12886-017-0667-0

**Published:** 2017-12-29

**Authors:** Monica Michelotti, Dirk F. de Korne, Jennifer S. Weizer, Paul P. Lee, Declan Flanagan, Simon P. Kelly, Anne Odergren, Sukhpal S. Sandhu, Charity Wai, Niek Klazinga, Aravind Haripriya, Joshua D. Stein, Melanie Hingorani

**Affiliations:** 1 Casey Eye Institute, Oregon Health and Sciences University, Portland, OR USA; 2 Singapore National Eye Centre, SingHealth Duke-NUS Academic Medical Centre, 11 Third Hospital Avenue, Singapore, 168751 Singapore; 3 Medical Innovation & Care Transformation, KK Women’s & Children’s Hospital, Singapore, Singapore; 4 Health Services & Systems Research, Duke-NUS Medical School, Singapore, Singapore; 5 Erasmus School of Health Policy & Management, Erasmus University Rotterdam, Rotterdam, Netherlands; 6 Department of Ophthalmology and Visual Sciences, W.K. Kellogg Eye Center, University of Michigan, Ann Arbor, USA; 7 Moorfields Eye Hospital NHS Foundation Trust, London, UK; 8 Department of Ophthalmology, Royal Bolton Hospital, Bolton, UK; 9 St Eriks Eye Hospital, Stockholm, Sweden; 10 The Royal Victorian Eye and Ear Hospital, Centre for Eye Research Australia, University of Melbourne, Melbourne, Victoria Australia; 11 Department of Social Medicine, Academic Medical Centre, University of Amsterdam, Amsterdam, Netherlands; 12 Cataract Services, Aravind Eye Care System, Chennai, India

**Keywords:** Outcomes, Measurement, Benchmarking, Quality improvement

## Abstract

**Background:**

To determine alignment of proposed international standard outcomes sets for ophthalmic conditions to metrics currently reported by eye hospitals.

**Methods:**

Mixed methods comparative benchmark study, including eight eye hospitals in Australia, India, Singapore, Sweden, U.K., and U.S. All are major international tertiary care and training centers in ophthalmology. Main outcome measure is consistency of ophthalmic outcomes measures reported.

**Results:**

International agreed standard outcomes (ICHOM) sets are available for cataract surgery (10 metrics) and macular degeneration (7 metrics). The eight hospitals reported 22 different metrics for cataract surgery and 2 for macular degeneration, which showed only limited overlap with the proposed ICHOM metrics. None of the hospitals reported patient reported visual functioning or vision-related quality of life outcomes measures (PROMs). Three hospitals (38%) reported rates for uncomplicated cataract surgeries only. There was marked variation in how and at what point postoperatively visual outcomes following cataract, cornea, glaucoma, strabismus and oculoplastics procedures were reported. Seven (87.5%) measured post-operative infections and four (50%) measured 30 day unplanned reoperation rates.

**Conclusions:**

Outcomes reporting for ophthalmic conditions currently widely varies across hospitals internationally and does not include patient-reported outcomes. Reaching consensus on measures and consistency in data collection will allow meaningful comparisons and provide an evidence base enabling improved sharing of “best practices” to improve eye care globally. Implementation of international standards is still a major challenge and practice-based knowledge on measures should be one of the inputs of the international standardization process.

**Electronic supplementary material:**

The online version of this article (doi: 10.1186/s12886-017-0667-0) contains supplementary material, which is available to authorized users.

## Background

Outcome reporting is mandated in many health care systems, and it is being incorporated into good medical practice and physician maintenance of certification [1, 2]. Several eye care providers and governmental entities indeed publicly report performance, often comparing outcomes with evidence-based benchmarks or targets [3–8]. The objective of reporting outcomes of high volume, high risk, or high cost procedures has been described as optimizing clinical efficacy and patient safety as well as cost-effectiveness [9–11].

Two leading subspecialties in ophthalmology recently developed a global standard outcomes set with the International Consortium of Outcomes Measurement (ICHOM) framework. The first one was for cataract [12, 13]. Cataract surgery, a removal of an opaque lens and replacement with an artificial intraocular lens implantation, is the most frequently performed elective surgical procedure in many countries, and rates of this surgery are likely to continue increasing as access improves in developing countries [14]. The second standard set was developed for macular degeneration, the leading cause of irreversible vision loss, accounting for over 15% of blindness in high-income countries, with an expected increase of the burden of disease [15, 16]. The standards sets propose to track preoperative visual acuity and target refraction, patient-reported visual function, intraoperative complications including capsule problems and dropped nucleus as well as other postoperative complications.

However, cataract surgery, macular degeneration and other subspecialties appear to currently have wide variations not only in outcome definition and risk stratification but also in metrics currently publicly reported. Collection of complete, reliable and robust outcome measurements is challenging [17, 18] and public reporting of individual surgeon’s outcomes in other surgical specialties, such as cardiac surgery, is not without controversy [19–21]. It is important to carefully select outcome measures and use unambiguous, consistent, transparent methodologies, to ensure that data can be reliably compared across institutions internationally [22].

Despite its effectiveness, rates of ophthalmic treatment vary substantially between countries and the need for systematic measurement of outcomes is paramount. One would expect standardized outcomes measures to deliver “value-based cataract care” as theoretically envisioned by Michael Porter and others [23, 24]. In recent years, the policy concept of value-based medicine, and a change in focus from ‘volume’ to ‘value’, have been increasingly discussed within in various health care systems, resulting in tracking of outcomes and changed reimbursement methodologies. Moreover, increased attention to surgical error, patient safety, and healthcare costs has increased public interest in reporting and comparing clinical performance. While the international standard sets are presented, little is known about the actual use of these indicators in hospital practice and the metrics that are currently used for other subspecialties.

In this paper, we map current ophthalmic outcome measures reported by a volunteer sample of major ophthalmic hospitals, which aspire to be exemplars of ophthalmic indicator use internationally to global standard sets, and provide insights into useful metrics to assess performance in ophthalmology and some of the challenges of implementing such measures across institutions.

## Methods

In this mixed methods descriptive study, we performed a review of the outcomes reported and compared this to existing standards [25]. Nine hospitals that are self-declared leaders in the routine publication and use of ophthalmic outcome indicators and that are members of an international eye hospital association were invited to participate in the study. The hospitals are participating in existing operational benchmarking initiatives that have been reported before [8, 26] and hence functioned as a convenient sample for the current review study on outcomes. Eight hospitals agreed on participation, and appointed one main point of contact that provided inside information on the availability and use of indicators in the respective hospitals. The websites of the eight hospitals were reviewed for publicly reported outcomes data or metrics on ophthalmic outcomes. Five institutions were found to have published data available on their website (Cole Eye Institute at Cleveland Clinic, University of Michigan W.K. Kellogg Eye Center, Massachusetts Eye and Ear Infirmary, Moorfields Eye Hospital, and Singapore National Eye Center). We searched the Medline, Cochrane, Emerald, Web of knowledge and Web of Science databases to find existing outcomes benchmark data using the following terms: ophthalmology, cataract, macular degeneration, outcomes, metrics, measures, and benchmarks. Preferred Practice Patterns from the American Academy of Ophthalmology (AAO), Clinical Guidelines from the Royal College of Ophthalmologists (RCOphth), and cited targets or benchmarks that each hospital had used were reviewed. In addition to the outcomes data published on the hospitals’ websites, non-published measures (eg, annual reports, departmental quality reports, auditing reports) were recorded through the main point of contact in the respective hospitals. Outcomes were added from Aravind Eye Hospital (Madurai, India), Moorfields Eye Hospital (London, United Kingdom), Singapore National Eye Center, The Royal Victorian Eye and Ear Hospital (Melbourne, Australia) and St. Erik’s Eye Hospital (Stockholm, Sweden). For the purposes of this paper, and to facilitate the sharing of non-publicly reported data, the hospital names were anonymized when comparing quality performance measures. University of Michigan’s and each of the participating hospitals’ Institutional Review Boards approvals were waived for this quality of care study.

## Results

Data from eight institutions were available and were grouped according to outcomes measures recommended in the international standard sets.

### Cataract surgery

Outcomes for cataract surgery are listed in Table 1, following the recommended classification of intraoperative and postoperative measures. Preoperative demographics, baseline visual status, ocular comorbidities and prior ophthalmic interventions are not listed. The eight hospitals reported a total of 22 different metrics for cataract surgery outcomes. None of the hospitals reported patient reported visual functioning. Three hospitals reported rates for uncomplicated cataract surgeries only. Six (75%) hospitals reported postoperative visual acuity, of which five (62.5%) reported postoperative outcomes of best-corrected visual acuity ≥ 20/40. There was marked variation in how and at what point postoperatively visual outcomes following cataract surgery were reported. Five (62.5%) reported the difference in refraction from the preoperative biometry based target. Seven (87.5%) measured post-operative infections and four (50%) measured 30 day unplanned reoperation rates. Seven institutions (87.5%) assessed visual acuity, and the most common outcome benchmark reported was best-corrected visual acuity of 20/40 or better. The most common refractive outcome measured was spherical equivalent within 1 diopter (D) of target refraction. With regard to postoperative complications, none of the hospitals reported on persistent corneal edema while only less then half of the hospitals reported other complications.

**Table 1:** Comparison of ICHOM outcome measures for cataract surgery and reported measures in study hospitals

	Item	Proposed ICHOM Measure	Reported Measure in Study Hospitals	Number of Hospitals Reporting	Reported Value
Intraoperative	Complications	Capsule problems	Posterior capsule rupture	6/8	0.64–2.11%
		Dropped nucleus or lens fragment into vitreous	Retained lens matter	1/8	0.21%
		Other	Intraoperative complications	1/8	1.87%
			Zonular dialysis	1/8	0.11%
			Choroidal hemorrhage	1/8	0.11%
Postoperative	Visual acuity	Post-operative visual acuity	BCVA 20/40 or better at 4–6 weeks, all cases	2/8	91%
			BCVA 20/40 or better at 4–6 weeks, excluding co pathology	3/8	87–100%
			1–14 letters improvement ETDRS VA score	1/8	≈ 52%
			≥ 15 letters improvement ETDRS VA score	1/8	≈ 42%
	Refractive error	Post-operative refractive error	Final spherical equivalent within 1 D of target	5/8	85–97%
	Patient-reported visual function	Rasch-calibrated score from Catquest 9SF or other Rasch-calibrated PROM	NA	0/8	NA
	Complications	Return to operating theater	Unplanned reoperation within 30 days of surgery	4/8	1.1–1.52%
		Endophthalmitis rate	Endophthalmitis rate	6/8	0.0–0.07%
		Persistent corneal edema	NA	0/8	NA
		Other (any postoperative complication within 3 months requiring treatment or compromising outcome)	Postoperative complications	1/8	0.36%
			Unplanned vitrectomy	4/8	0.48–1.93%
			Iris trauma	1/8	0.32%

*ETDRS* Early Treatment Diabetic Retinopathy Study, *BCVA* best corrected visual acuity, *D* Diopter, *VA* visual acuity

### Macular degeneration

Outcomes for macular degeneration are presented in Table 2, following the order of the standard set visual functioning and vision-related quality of life, disutility of care and disease control. Two hospitals (25%) reported on visual acuity. None of the hospitals reported on mobility and independence, emotional well-being or reading and accessing information. For complications of treatment, the most common measure was endophthalmitis rate following intravitreal injection, presented by five hospitals (62.5%). None of the hospitals reported the presence of intraretinal or subretinal fluid or hemorrhage.

**Table 2:** Comparison of ICHOM outcome measures for macular degeneration and reported measures in study hospitals

	Item	Propose ICHOM Measure	Reported measure in study hospitals	Number of hospitals reporting	Target	Reported value
Visual functioning and vison-related quality of life	Distance visual acuity	Distance visual acuity (best of uncorrected, corrected, or pinhole) in the affected eye.	Gain VA (15 ETDRS letters) after injections for macular degeneration	1/8	> 20%	20.7%
			Visual stability (loss < 15 ETDRS letters) after injections for macular degeneration	1/8	> 80%	90.25%
	Mobility and independence	Impact of vision impairment questionnaire	NA	0/8	None	NA
	Emotional well-being	Impact of vision impairment questionnaire	NA	0/8	None	NA
	Reading and accessing information	Impact of vision impairment questionnaire	NA	0/8	None	NA
Disutility of care	Number of treatments	Documentation of individual treatments received for macular degeneration	Appointment access and check in (% best response)	1/8	None	≈ 63%
			Clinic wait times and comfort (% best response)	1/8	None	≈ 45%
	Complications of treatment	Endophthalmitis: severe intraocular inflammation within 3 months of last intraocular treatment	Endophthalmitis after anti-VEGF intravitreal injections	5/8	None or 0.2–1.9%^a^ 0.05% (MARINA)	0–0.18%
Disease control	Presence of fluid, edema, or hemorrhage	Presence of intraretinal or subretinal fluid or hemorrhage that is attributable to activity of the neovascular lesion	NA	0/8	None	NA

*ETDRS* Early Treatment Diabetic Retinopathy Study, *RD* retinal detachment, *VEGF* vascular endothelial growth factor, *PVR* proliferative vitreo-retinopathy

^a^Bhavsar et al. Risk of endophthalmitis after intravitreal drug injection when topical antibiotics are not required. Arch Ophthalmol 2009; 127(12): 1581–1583

### Other outcomes

The outcomes of the other ophthalmic procedures reported by the hospitals are presented as Supplementary Data. Refractive surgery outcomes are presented in Additional file 1. Several hospitals report refractive outcomes in detail, which is useful for marketing purposes to potential customers/consumers for such procedures. However, there are many permutations and combinations of refractive treatments and preoperative refractive errors precluding easy comparison. Medical and surgical retina outcome measures other than related to macular degeneration are shown in Additional file 2. Some institutions risk-stratify surgical retina outcomes by initial surgery or reoperations, while others subdivide cases by specific etiology. Metrics include both functional (visual acuity) and structural (retinal re-attachment rate) outcomes. There is variation in reported outcome measures for pediatric and strabismus surgery, Additional file 3. Some hospitals measure complications or reoperation rates. There are also two hospitals that measure the postoperative improvement in ocular alignment, with success being defined as less than 10–15 prism diopter of residual eso- or exotropia. Additional file 4 demonstrates glaucoma and corneal surgery outcome measures. The cornea outcomes primarily focus on corneal transplant failure or rejection rate. The expected failure rate differs significantly based on which type of corneal graft, and some institutions differentiate their results based on corneal procedure type. Some hospitals report post-operative visual acuity or improvement following corneal transplant surgery. All institutions measure either intraoperative or postoperative complications with some reporting glaucoma surgery ‘failure’ or ‘success’ rates and/or post-operative endophthalmitis rates. The latter is presumed to be early surgery-related endophthalmitis, with little information regarding late blebitis related cases. Two institutions set a goal for intraocular pressure (IOP) of < 17 mmHg or < 21 mmHg and one institution looked at only the change in IOP following surgery. Interestingly, data is not published on the use of anti-metabolites (including the re-needling rates with/without antimetabolites) and whether surgery is combined with cataract. Oculoplastics outcome measures are shown in Additional file 5. Some departments measure reoperation rates or complication rates. Two hospitals measure postoperative ptosis repair success – one based on postoperative eyelid symmetry and the other looks at patient satisfaction rates on a scale of 1–10.

## Discussion

We present the first review of ophthalmic outcome measures reported by eye hospitals in diverse populations in various nations. The study is limited by the number of institutions who publicly report outcomes and indicators, and a reluctance from a number of institutions to either devote resource to gather regular indicator results to benchmark or, if gathered, to share or publish indicator results. This rendered a comprehensive or global data gathering study impractical at this time, but the use of a sample of leading institutions was possible and, although by necessity only permitting descriptive statistics, does demonstrate both the utility and the issues in attempting to use ophthalmic indicators to benchmark and compare performance across institutions and countries. Although several hospitals report similar outcomes and targets or benchmarks in each subspecialty, there is little congruence on which outcomes or benchmarks should be reported, which methodologies should be used and how to address preoperative risk and co-morbidity. Despite the existence of two internationally agreed ICHOM standards for eye care, compliance with the proposed measures is limited and especially measurement of recommended patient reported visual functioning or vision-related quality of life outcomes measures (PROM’s) is not yet taking place systematically. While we realize that health care systems are complex and (large) differences between health systems could be barrier for valid comparisons, [8] other surgical specialties have long recorded outcomes and such reporting has improved clinical and cost effectiveness as well as patient safety [19, 27]. Initial fears of surgeon avoidance of high-risk cases or unfair reputational damage have proved largely unfounded [19]. In the U.K. many surgical specialties publish their outcomes, and in 2016 the first pilot with a national reporting on cataract surgery outcomes was done in a similar fashion [28]. Pay for performance tools have been instituted by Medicare in the US. The Physician Quality Reporting System (PQRS) was initially started as an incentive system to promote quality of care and outcome reporting. In 2017, ophthalmic practices who do not report three quality measures 50% of time will receive a 2 % penalty on Medicare reimbursements 2 years down the road. Physicians who report nine measures in three of the new national quality strategy domains will receive a 0.5% bonus payment [29]. With the increasing prevalence of electronic record systems, quality-focused healthcare, rising patient expectations as well as increasing cost pressures, our expectation is that outcome reporting in ophthalmology will globally become the norm instead of the exception.

Goals in clinical outcome reporting include encouraging quality improvement, creating a minimum standard for providers, driving innovation in care, performance management of units and individual surgeons, distributing pay incentives, increasing informed decision making for patients as well as promoting public confidence in healthcare providers, and contributing to research [30, 31]. Reporting outcomes data to the public may promote greater scrutiny of health care and reduce variations in the quality of care, thereby making physicians more accountable [32].

One potentially straightforward method of outcome reporting is to indicate ophthalmic quality and safety by measurement of the rate of serious adverse healthcare associated events such as postoperative infections, unplanned reoperations, and so called ‘never events’. ‘Never events’ are serious, potentially preventable errors in healthcare, for example in cataract care these may include operating on the wrong patient, on the wrong eye, or inserting the incorrect (unplanned) intraocular lens [33]. ‘Never events’ are taken seriously and investigated with root cause analysis, and actions should be taken to prevent error recurring with a culture of fair blame. There was significant consensus in this area, with all institutions reporting similar values; however some institutions reported only subspecialty-specific data.

In cataract surgery, many institutions report visual acuity – the most common target was best-corrected visual acuity of 20/40 or better, however another strategy is to consider improvement in visual acuity. We found that timing of follow-up for outcome reporting following cataract surgery may be influenced by particular health system or hospital’s practice patterns. For example if post-operative cataract patients are discharged from ophthalmic care or followed up in the community the return of outcome data may be problematic.

It is notable that there is a lack of hospitals using patient-reported measures, while patient satisfaction and experience rates are commonly used measure by payers and state agencies. In contrast, while a variety of instruments have been employed in research studies for assessing the impacts of cataract (and other ocular) surgery on patients’ symptoms, functional ability, wellbeing and health, these are not yet generally publicly reported in clinical care. These PROM’s can be divided into generic assessments that have been designed to apply across a range of different health conditions (for example, the EuroQol EQ-5D and SF-36) and instruments that focus specifically on vision-related conditions (for example, the VF-14). Properly developed PROM’s are valid and reliable research tools but they can be cumbersome for patients to use routinely on a large-scale basis [34]. The RCOphth resisted the use of the VF-14 PROM metrics for routine use in the U.K. National Health Services (NHS) cataract surgery for referral or reimbursement purposes, as the College was of the opinion that it added no value in routine NHS care in the U.K. [1]. Subsequent research confirmed the College’s concerns about the VF-14 tool [35, 36]. PROM’s tools are increasingly being tied to reimbursement and their use is likely to be an area of growth in ophthalmic care the future.

### Limitations of current ophthalmic indicator use

As electronic records become standardized and more patient specific data is readily available, ophthalmic outcome measures will become easier to obtain and potentially more meaningful. The merit of quality reporting depends on data quality, risk adjustment, sample size, and the specification of quality measures themselves [37]. Current ophthalmic outcomes reporting methods do not usually take into account the complicated statistical methods for risk adjustment used in other healthcare fields and hence we have not applied any quantitative comparison or statistical tests in this study. The issue of case mix adjustments is a critical topic in other specialties that publicly report outcomes. Failure to adjust for such may discourage surgeons from treating high-risk patients and therefore deny high-risk patients the opportunity of benefiting from surgery, while appropriate risk adjustment can do much to allay these concerns. There has been reported reluctance of some cardiac surgeons to operate on high-risk patients for these reasons [38, 39] and, without appropriate risk adjustment, unintended distortions of appropriate surgical care may thus arise to the ultimate detriment of the public [40].

At present, hierarchical regression is the gold standard for risk adjustment of outcomes and for producing provider report cards; however this gold standard is rarely used [41] and no equivalent modeling has been developed in ophthalmology to date. The National Ophthalmic Database (NOD) in the U.K. has compiled a national cataract data set, and participating cataract surgeons have the ability to compare their individual surgical data to others in a risk-adjusted manner [28]. A new American registry [42] may have the ability to risk stratify eye surgeons’ case mix, while previous groups have either excluded any patients with comorbidities or not performed case mix analysis.

### Next steps

Increasingly, there are national and multi-national collaborations that are collecting large quantities of data on structures, processes, and outcomes of care, and this is facilitated by increasing use of electronic health records which removes many previous barriers to large scale data gathering. These databases will become more prominent and powerful with their ability to retrieve data directly from electronic medical records.

The European Registry of Quality Outcomes for Cataract and Refractive Surgery (EUREQUO) was initiated in 2008 by 11 European countries to improve treatment and standards of care for cataract and refractive surgery and to develop evidence based guidelines [43]. As of mid-2016, more than 2 million cataract operations were included in the database and specific guidelines were established regarding cataract surgery. These guidelines, in addition to the benchmark set by Hahn and colleagues [44] for cataract surgery, may be useful benchmarks for other organizations to use.

The Intelligent Research in Sight (IRIS) registry is a clinical data registry designed by the American Academy of Ophthalmology (AAO) that opened to all AAO member physicians since 2014. This registry automatically collects data from participating electronic health record systems and provide benchmark reports for participating practitioners. As of 1 January 2016, the IRIS registry included 11,739 physicians, and registered 72.05 million visits representing 20.5 million patients presumably [45, 46].

Finally, ICHOM’s standard guideline sets for cataract surgery [13] and macular degeneration [15] have been rigorously developed with the goal of improving quality of health care, reducing health care costs, and supporting informed decision-making. As the currently reported outcomes were found to be only partially in line with the standards, in some instances the ICHOM sets might need to shift to align with what is currently done. In most instances, however, institutions might opt to align their reporting with the standard sets, which is the direction the institutions participating in our study are currently taking.

Our study shows that much more alignment on the concept of value based eye care will be needed to implement the standards in the day-to-day hospital practice and gives suggestions for future metrics in other subspecialties to be developed. Practice-based knowledge on outcome measures, as provided in this paper, should form part of the input of the international standardization process to assure implementation.

## Conclusion

Outcomes reporting for ophthalmic surgery currently widely varies across hospitals globally, and value too often seems to be defined in the eyes of the beholder. Reaching consensus on outcomes measures will allow meaningful comparisons of outcomes at different hospitals which will provide an evidence base enabling improved sharing of “best practices” to improve eye care globally. Identifying standardized and common metrics is an important first step to improve the quality of outcome data. As data improves and methods of risk adjustment become more mature, outcome metrics can help institutions to improve the quality of the patient care they provide and demonstrate this quality to patients, payers of healthcare and regulators. However, setting of international standards on outcomes should include practice-based knowledge, as provided through the mapping exercise in this paper, from the beginning.

## Additional files

Additional file 1: Refractive and corneal outcomes reported by the hospitals. Description of date: 32 refractive and corneal outcomes reported by the hospitals. (DOCX 18 kb)

Additional file 2: Retinal outcomes reported by the hospitals. Description of data: 35 retinal outcomes reported by the hospitals. (DOCX 19 kb)

Additional file 3: Pediatrics and strabismus outcomes reported by the hospitals. Description of data: 15 Pediatrics and strabismus outcomes reported by the hospitals. (DOCX 17 kb)

Additional file 4: Cornea and glaucoma outcomes reported by the hospitals. Description of data: 29 cornea and glaucoma outcomes reported by the hospitals. (DOCX 20 kb)

Additional file 5: Oculoplastics outcomes reported by the hospitals. Description of the data: 11 oculoplastics outcomes reported by the hospitals. (DOCX 16 kb)

## Abbreviations

AAO: American Academy of Ophthalmology
EQ-5D: EuroQual 5D
EUREQUO: European Registry of Quality Outcomes for Cataract and Refractive Surgery
ICHOM: International consortium for health outcomes measurement
IOP: Intraocular pressure
IRIS: Intelligent Research in Sight
NHS: National Health Services
NOD: National Ophthalmic Database
PQRS: Physician Quality Reporting System
PROM: Patient reported outcomes measure
SF-36: 36-item short form survey
VF-14: Visual functioning index 14
WAEH: World Association of Eye Hospitals
