# Supplementary material for: Mapping standard ophthalmic outcome sets to metrics currently reported in eight eye hospitals
Source: BMC Ophthalmol. 2017 Dec 29;17:269. doi: 10.1186/s12886-017-0667-0 (PMC5747118; doi:10.1186/s12886-017-0667-0)
Supplement: Supplementary file 1 — Refractive and corneal outcomes reported by the hospitals. Description of date: 32 refractive and corneal outcomes reported by the hospitals. (DOCX 18 kb) [file 12886_2017_667_MOESM1_ESM.docx]

**Additional file 1**: Refractive outcomes reported by the hospitals

|  | **Metric** | **Target** | **Reported Value** |
| --- | --- | --- | --- |
| **Hospital A*** |  |  |  |
|  | Femto LASIK: Refractive predictability within ±0.5D | None | 97% |
|  | Femto LASIK: UCVA of 20/16 or better | None | 75% |
|  | Femto LASIK: UCVA of 20/20 or better | None | 94% |
|  | Femto LASIK: UCVA of 20/25 or better | None | 100% |
|  | Distance only LASIK for high myope (>7D) with cyl <3D): Refractive predictability within ±0.5D | None | 82% |
|  | Distance only LASIK for high myope (>7D) with cyl <3D): UCVA of 20/20 or better | None | 86% |
|  | Distance only PRK for myopia: Refractive predictability within ±0.5D | None | 97% |
|  | Distance only PRK for myopia: UCVA of 20/20 or better | None | 97% |
|  | Distance only LASIK for hyperopia: Refractive predictability within ±0.5D | None | 86% |
|  | Distance only LASIK for hyperopia: UCVA of 20/20 or better | None | 86% |
| **Hospital B** |  |  |  |
|  | Refractive surgery: UCVA 20/40 or better at 3 months | 90%^[[1]](#endnote-1)^ | 100% |
|  | Refractive surgery: UCVA 20/20 or better at 3 months | 60% | 88% |
| **Hospital D** |  |  |  |
|  | LASIK for low myopia: within 0.5D of target (spherical equivalent) | >85% | 88.7% |
|  | Loss of ≥2 lines VA after LASIK | <1% | 0% |
|  | LASIK for low myopia: UCVA 20/30 or better | ≥80% | 87.9% |
| **Hospital C** |  |  |  |
|  | LASIK retreatment rate within 6 months | 5-28%^[[2]](#endnote-2)^ | 7.4% |
|  | Low myopia: within 0.5 D of target | None | 97.3% |
|  | Moderate myopia: within 0.5 D of target | None | 82.1% |
|  | High myopia: within 0.5 D of target | None | 96.8% |
|  | Hyperopia: within 0.5 D of target | None | 68.0% |
|  | LASIK for myopia: within 0.5D of target (spherical equivalent) | 68-83%^[[3]](#endnote-3)^ ^[[4]](#endnote-4)^ ^[[5]](#endnote-5)^ | 88.1% |
|  | LASIK for hyperopia: within 0.5D of target (spherical equivalent) | 53.2%-91%^[[6]](#endnote-6)^ ^[[7]](#endnote-7)^ | 68% |
| **Hospital H** |  |  |  |
|  | Femto LASIK: UCVA of 6/4.5 or better | None | 16.0% |
|  | Femto LASIK: UCVA of 6/6 or better | None | 83.1% |
|  | Femto LASIK: within 0.5D of target | None | 88.3% |
|  | UCVA 6/12 or better | None | 99.5% |
|  | UCVA 6/6 or better | None | 83.1% |
|  | UCVA 6/4.5 or better | None | 16.0% |
|  | Efficacy Index: Ratio of postoperative UCVA and preoperative BCVA | None | 0.98 |
|  | Within 1.0D of target | None | 98.6% |
|  | Within 0.5D of target | None | 88.3% |
|  | Safety Index: Ratio of postoperative and preoperative BCVA | None | 1.14 |

* Additional values available for hospital A, too extensive to include in table. LASIK = Laser-Assisted in situ Keratomileusis, UCVA = uncorrected visual acuity, BCVA = best corrected visual acuity, PRK = photorefractive keratectomy,

1. Schallhorn SC, Farjo AA, Huang D, Boxer Wachler BS, Trattler WB, Tanzer DJ, Majmudar PA, Sugar A; American Academy of Ophthalmology. Wavefront-guided LASIK for the correction of primary myopia and astigmatism a report by the American Academy of Ophthalmology. Ophthalmology. 2008 Jul;115(7):1249-61. doi: 10.1016/j.ophtha.2008.04.010. [↑](#endnote-ref-1)
2. Hersh PS, Fry KL, Bishop DS. Incidence and associations of retreatment after LASIK. Ophthalmology 2003; 110(4): 748-754. [↑](#endnote-ref-2)
3. Bailey MD and Zadnick K. Outcomes of LASIK for myopia with FDA-approved lasers. Cornea 2007; 26(3), 246- 254 [↑](#endnote-ref-3)
4. Yuen LH, Chan WK, Koh J, Mehta JS, Tan DT. A 10-year prospective audit of LASIK outcomes for myopia in 37,932 eyes at a single institution in Asia. Ophthalmology 2010; 117(6): 1236–1244. 3 [↑](#endnote-ref-4)
5. Mrochen M, Kaemmerer M, Seiler T. Clinical results of wavefront-guided laser in situ keratomileusis 3 months after surgery. J Cataract Refract Surg 2001; 27(2): 201–207 [↑](#endnote-ref-5)
6. Jaycock PD, O’Brart DPS, Rajan MS, Marshall J. 5-year follow-up of LASIK for hyperopia. Ophthalmology 2005; [↑](#endnote-ref-6)
7. Keir NJ, Simpson T, Hutchings N, Jones L, Fonn D. Outcomes of wavefront-guided laser in situ keratomileusis for hyperopia. J Cataract Refract Surg 2011; 37(5): 886–893. [↑](#endnote-ref-7)
