# Supplementary material for: Mapping standard ophthalmic outcome sets to metrics currently reported in eight eye hospitals
Source: BMC Ophthalmol. 2017 Dec 29;17:269. doi: 10.1186/s12886-017-0667-0 (PMC5747118; doi:10.1186/s12886-017-0667-0)
Supplement: Supplementary file 2 — Retinal outcomes reported by the hospitals. Description of data: 35 retinal outcomes reported by the hospitals. (DOCX 19 kb) [file 12886_2017_667_MOESM2_ESM.docx]

**Additional file 2**: Retinal outcomes reported by the hospitals

|  | **Metric** | **Number of Institutions Reporting** | **Target** | **Reported Value** |
| --- | --- | --- | --- | --- |
| **Retinal detachment repair** | Retinal reattachment 30 days postoperatively | 1/8 | 80%^[[1]](#endnote-1)^ | 90% |
|  | ≥ 2 lines vision improvement | 1/8 | 50.80% |  |
|  | Intraoperative complications | 2/8 | None | 1.6-2.2% |
|  | Postoperative complications | 1/8 | None | 3% |
|  | Reoperations for retinal detachment repair within 30 days of original repair | 1/8 | 0 | 5.7% |
| **Primary RD** | Retina reattached with 1 surgery | 3/8 | None, >75% | 87.1-89.4% |
|  | Final retinal reattachment at 6 months | 2/8 | None, or 97-100%^[[2]](#endnote-2)^ ^[[3]](#endnote-3)^ ^[[4]](#endnote-4)^^[[5]](#endnote-5)^ ^[[6]](#endnote-6)^ | 96.9-98.9% |
|  | Final success of retinal detachment surgery at 6 month (Posterior pole flat, Peripheral Retina Off) | 1/8 | None | 99.0% |
|  | Failure of RD surgery | 1/8 | None | 2.1% |
|  | Mean VA improvement | 1/8 | None | 14.8 ETDRS letters |
|  | Gain ≥ 3 lines VA | 1/8 | None | 40% |
| **Complicated RD (Recurrent, giant tear, or PVR)** | Reattachment rate | 1/8 | None | 97% |
| **PVR RD repair** | Mean VA improvement | 1/8 | None | 15.2 ETDRS letters |
|  | Gain ≥ 3 lines VA | 1/8 | None | 35% |
|  | Loss ≥ 3 lines VA | 1/8 | None | 8.8% |
| **Macular Hole** | Anatomic closure | 2/8 | None | 90.4-100% |
|  | Anatomic closure 1 operation | 2/8 | >80% | 80.6-86.2% |
|  | Success at 6 months  (Hole edge flat but not closed) | 1/8 | None | 93.6% |
|  | Failure of macular hole surgery | 1/8 | None | 3.2% |
|  | Gain ≥ 3 lines VA | 2/8 | None | 53-54.3% |
|  | Mean improvement VA | 1/8 | None | 17.4 ETDRS letters (≥3 lines) |
|  | Intraoperative Complications | 1/8 | None | 2.1% |
| **Intravitreal injections** | Endophthalmitis after anti-VEGF intravitreal injections | 5/8 | None or 0.2-1.9%^[[7]](#endnote-7)^  0.05% (MARINA) | 0-0.18% |
|  | Gain VA (15 ETDRS letters) after injections for macular degeneration | 1/8 | >20% | 20.7% |
|  | Visual stability (loss <15 ETDRS letters) after injections for macular degeneration | 1/8 | >80% | 90.25% |
| **ERM** | Mean VA improvement | 1/8 | None | 11.2 ETDRS letters |
|  | Gain ≥ 3 lines VA | 1/8 |  | 22% |
| **Diabetic Vitrectomy** | Tractional RD: Mean improvement in VA | 1/8 | None | 11.9 ETDRS letters |
|  | Tractional RD: Gain ≥ 3 lines VA | 2/8 | None | 36-55% |
|  | Tractional RD: Loss ≥ 3 lines VA | 2/8 | None | 0% - “1 patient” |
|  | Vitreous Hemorrhage: Mean improvement in VA | 1/8 | None | 37.4 ETDRS letters |
| **Other** | Time from referral to assessment of proliferative diabetic retinopathy | 1/8 | 80% | 90.3% |
|  | Posterior capsule rupture in vitrectomised eyes | 1/8 | <5% | 1.6% |
|  | Retinopexy cryotherapy: success at 6 months for prevention of RD: | 1/8 | None | 95% |
|  | Retinopexy laser: success at 6 months for prevention of RD | 1/8 | None | 90% |

ETDRS = Early Treatment Diabetic Retinopathy Study, RD = retinal detachment, VEGF = vascular endothelial growth factor, PVR = proliferative vitreo-retinopathy

1. Thompson JA, Snead MP, Billington BM, Barrie T, Thompson JR, Sparrow JM. National audit of the outcome of primary surgery for rhegmatogenous retinal detachment. II. Clinical outcomes. Eye (Lond). 2002 Nov;16(6):771-7. [↑](#endnote-ref-1)
2. Han DP, Mohsin NC, Guse CE, Hartz A, Tarkanian CN, Southeastern Wisconsin Pneumatic Retinopexy Study Group. Comparison of pneumatic retinopexy and scleral buckling in the management of primary rhegmatogenous retinal detachment. Am J Ophthalmol 1998; 126(5), 658-668. 2 [↑](#endnote-ref-2)
3. Avitabile T, Bartolotta G, Torrisi B, Reibaldi A. A randomized prospective study of rhegmatogenous retinal detachment cases treated with cryopexy versus frequency-doubled nd:yag laser-retinopexy during episcleral surgery. Retina 2004; 24(6), 878-882. 3 [↑](#endnote-ref-3)
4. Azad RV, Chanana B, Sharma YR, Vohra R. Primary vitrectomy versus conventional retinal detachment surgery in phakic rhegmatogenous retinal detachment. Acta Ophthalmol Scand 2007; 85, 540-545 [↑](#endnote-ref-4)
5. Sullivan PM, Luff AJ, Aylward GW. Results of primary retinal reattachment surgery: a prospective audit. Eye 1997; 11, 869-871. [↑](#endnote-ref-5)
6. Sullivan PM, Luff AJ, Aylward GW. Results of primary retinal reattachment surgery: a prospective audit. Eye 1997; 11, 869-871. [↑](#endnote-ref-6)
7. Bhavsar et al. Risk of endophthalmitis after intravitreal drug injection when topical antibiotics are not required. Arch Ophthalmol 2009; 127(12): 1581-1583 [↑](#endnote-ref-7)
