# Supplementary material for: Mapping standard ophthalmic outcome sets to metrics currently reported in eight eye hospitals
Source: BMC Ophthalmol. 2017 Dec 29;17:269. doi: 10.1186/s12886-017-0667-0 (PMC5747118; doi:10.1186/s12886-017-0667-0)
Supplement: Supplementary file 3 — Pediatrics and strabismus outcomes reported by the hospitals. Description of data: 15 Pediatrics and strabismus outcomes reported by the hospitals. (DOCX 17 kb) [file 12886_2017_667_MOESM3_ESM.docx]

**Additional file 3** Pediatrics and strabismus outcomes reported by the hospitals

|  | **Metric** | **Institution** | **Target** | **Reported Value** |
| --- | --- | --- | --- | --- |
| **Strabismus correction unspecified** | Unplanned strabismus reoperations within 90 days of original surgery | 1/8 | 0 | 0.9% |
|  | Serious complications strabismus surgery | 1/8 | <2.2% | 0.3% |
|  | Correction to ≤15 PD of residual ET at 3 months | 1/8 | 60%^[[1]](#endnote-1)^ | 100% |
|  | Outcome of ET10-XT10PD, HT<5PD or attain cosmesis at 1 year or last follow-up if earlier | 1/8 | None | 83% |
|  | Achieve occasional or no diplopia at 1 year or last follow-up if earlier | 1/8 | None | 86% |
|  | Achieve better or normal head posture at 1 year or last follow-up if earlier | 1/8 | None | 83% |
| **Adult strabismus correction** | Adult correction: Poor (over or under corrected) | 1/8 | None | 27% |
|  | Adult correction: Good (alignment within 10 PD if no diplopia, and/or diplopia disappeared, and/or anomalous head position resolved) | 1/8 | None | 73% |
| **Pediatric strabismus correction** | Pediatric correction: Poor (over or under corrected) | 1/8 | None | 10% |
|  | Pediatric correction: Good (constant deviation< 10PD in primary position and/ or anomalous head position resolved) | 1/8 | None | 90% |
| **Pediatric cataract** | Pediatric developmental cataract: VA of 6/18 or better | 1/8 | None | 82.5% |
|  | Pediatric congenital cataract: VA of 20/60 or better | 1/8 | None | 63.6% |
|  | Ectropion surgery success at 1 year | 1/8 | >80% | 100% |
| **Other** | Premature baby eye retinopathy of prematurity screening compliance | 1/8 | 99% | 100% |
|  | Success of probing for congenital tear duct blockage | 1/8 | >85% | 86% |

PD = prism Diopter, ET = esotropia XT = Exotropia, HT = hypotropia

1. Jatla KK, Enzenauer RW. Strabismus outcomes/quality control: the application of statistical process control (SPC) to one muscle and two muscle simple horizontal strabismus of 25 PD or less. Binocul Vis Strabismus Q. 2006;21(4):215-22. [↑](#endnote-ref-1)
