# Supplementary material for: Mapping standard ophthalmic outcome sets to metrics currently reported in eight eye hospitals
Source: BMC Ophthalmol. 2017 Dec 29;17:269. doi: 10.1186/s12886-017-0667-0 (PMC5747118; doi:10.1186/s12886-017-0667-0)
Supplement: Supplementary file 4 — Cornea and glaucoma outcomes reported by the hospitals. Description of data: 29 cornea and glaucoma outcomes reported by the hospitals. (DOCX 20 kb) [file 12886_2017_667_MOESM4_ESM.docx]

**Additional file 4**: Cornea and glaucoma outcomes reported by the hospitals

|  | **Metric** | **Number of Institutions Reporting** | **Target** | **Reported Value** |
| --- | --- | --- | --- | --- |
| **All grafts or not specified** | Corneal graft clear at 6 months | 1/8 | 70% | 86.7% |
|  | Graft rejection (at 1 year) | 2/8 | None | 4.0-4.8% |
|  | Intraoperative complications | 1/8 | 0 | 1.2% |
|  | Postoperative complications | 1/8 | 0 | 5.7% |
|  | Refraction within 1D of target | 1/8 | None |  |
| **PK** | PK failure (graft not clear) at 1 year | 1/8 | None | 8.5% |
|  | PK graft survival or graft clear (optical cases) at 1 year  PK graft clear at 3-6 months | 2/8  2/8 | None,  92.5-95% | 83.3-91.6%  89-92.8% |
|  | PK mean improvement in VA | 1/8 | None | 38 ETDRS letters |
| **DSAEK** | DSAEK graft clear at 3-12 months | 2/8 | None | 92.1%-99% |
|  | DSAEK failure (graft not clear) at 1 year | 1/8 | <12% | 8.9% |
|  | DSAEK mean improvement in VA | 1/8 | None | 28.49 ETDRS letters |
| **DALK** | DALK graft failure at 1 year | 1/8 | None | 6.7% |
|  | Clear DALK graft at 1 year  DALK 1 year graft survival (optical cases) | 2/8 | None | 93.3-96% |
| **KPro** | VA 20/200 or better after keratoprosthesis | 1/8 | 56-89% | 66.7% |
|  | Keratoprosthesis retention rate | 1/8 | 90.5-95% | 100% |
| **Tube and trabeculectomy** | Intraoperative complications | 2/8 | 0  ^[[1]](#endnote-1)^ ^[[2]](#endnote-2)^ ^[[3]](#endnote-3)^ ^[[4]](#endnote-4)^ | 0.04-0.4% |
|  | Postoperative complications at 3 months | 1/8 | 0 | 3.5% |
|  | Endophthalmitis after tube or trabeculectomy surgery | 1/8 | 0.12-8.33%^[[5]](#endnote-5)^ | 0% |
|  | IOP ≤ 17 at 6 months postoperatively or with plan to achieve IOP lowering | 1/8 | 70%^[[6]](#endnote-6)^ ^[[7]](#endnote-7)^ ^[[8]](#endnote-8)^ ^[[9]](#endnote-9)^ | 100% |
|  | Mean IOP at 6 months | 1/8 | None | 11.4 mmHg |
| **Trabeculectomy** | IOP change after trabeculectomy | 1/8 | 0 | 21 to 15.5 |
|  | Failure (IOP≥21 with or without anti-glaucoma medication or further anti-glaucoma procedure) at 1 year | 1/8 | 15% | 8.20% |
|  | Success (IOP≤21 with or without anti-glaucoma medication) at 1 year | 1/8 | None | 92.2% |
| **Tube** | IOP change after tube surgery | 1/8 | 0 | 25.8 to 16.1 |
|  | Glaucoma tube drainage surgery failure (IOP≥21 with or without anti-glaucoma medication or further anti-glaucoma procedure) at 1 year | 1/8 | <10% | 8% |
|  | Posterior capsule rupture in glaucoma patients | 1/8 | 0 | 1.04% |
|  | Seton implant surgery: IOP≤21 with or without anti-glaucoma medication at 1 year | 1/8 |  | 97.3% |
| **Trabeculecomy and cataract surgery** | Trabeculectomy and cataract surgery: IOP≤21 with or without anti-glaucoma medication at 1 year | 1/8 | None | 97.5% |

PK = penetrating keratoplasty; DSAEK = Descemet's stripping automated endothelial keratoplasty, DALK = deep anterior lamellar keratoplasty, D = Diopter, ETDRS = Early Treatment Diabetic Retinopathy Study, KPro-= keratoprosthesis, IOP = Intraocular Pressure (mmHg)

1. Ang GS, Varga Z, Shaarawy T. Postoperative infection in penetrating versus non-penetrating glaucoma surgery. Br J Ophthalmol 2010; 94(12): 1571-1576. [↑](#endnote-ref-1)
2. Jampel HD, Musch DC, Gillespie BW, Lichter PR, Wright MM, Guire KE. Perioperative complications of trabeculectomy in the Collaborative Initial Glaucoma Treatment Study (CIGTS). Am J Ophthalmol 2005; 140(1): 16-22. 3 [↑](#endnote-ref-2)
3. Gedde SJ, Herndon LW, Brandt JD, Budenz DL, Feuer WJ, Schiffman JC. Surgical complications in the tube versus trabeculectomy study during the first year of follow-up. Am J Ophthalmol 2007; 143(1): 23-31. 4 [↑](#endnote-ref-3)
4. Christakis PG, Tsai JC, Zurakowski D, Kalenak JW, Cantor LB, Ahmed IK. The Ahmed Versus Baerveldt Study: design, baseline patient characteristics, and intraoperative complications. Ophthalmology 2011; 118(11): 2172-2179. [↑](#endnote-ref-4)
5. Ang GS, Varga Z, Shaarawy T. Postoperative infection in penetrating versus non-penetrating glaucoma surgery. Br J Ophthalmol 2010; 94(12): 1571-1576. [↑](#endnote-ref-5)
6. Wong TT, Khaw PT, Aung T, Foster PJ, Htoon HM, Oen FT, Gazzard G, Husain R, Devereux JG, Minassian D, Tan SB, Chew PT, Seah SK. The singapore 5-Fluorouracil trabeculectomy study: effects on intraocular pressure control and disease progression at 3 years. Ophthalmology. 2009 Feb;116(2):175-84. doi: 10.1016/j.ophtha.2008.09.049. [↑](#endnote-ref-6)
7. Palanca-Capistrano AM, Hall J, Cantor LB, Morgan L, Hoop J, WuDunn D.

   Long-term outcomes of intraoperative 5-fluorouracil versus intraoperative mitomycin C in primary trabeculectomy surgery. Ophthalmology. 2009 Feb;116(2):185-90. doi: 10.1016/j.ophtha.2008.08.009. Epub 2008 Oct 18. [↑](#endnote-ref-7)
8. Lichter PR, Musch DC, Gillespie BW, Guire KE, Janz NK, Wren PA, Mills RP; CIGTS Study Group. Interim clinical outcomes in the Collaborative Initial Glaucoma Treatment Study comparing initial treatment randomized to medications or surgery. Ophthalmology. 2001 Nov;108(11):1943-53. [↑](#endnote-ref-8)
9. Bahar I, Kaiserman I, McAllum P, Slomovic A, Rootman D. Comparison of posterior lamellar keratoplasty techniques to penetrating keratoplasty. Ophthalmology. 2008 Sep;115(9):1525-33. doi: 10.1016/j.ophtha.2008.02.010. Epub 2008 Apr 28. [↑](#endnote-ref-9)
