# Supplementary material for: Mapping standard ophthalmic outcome sets to metrics currently reported in eight eye hospitals
Source: BMC Ophthalmol. 2017 Dec 29;17:269. doi: 10.1186/s12886-017-0667-0 (PMC5747118; doi:10.1186/s12886-017-0667-0)
Supplement: Supplementary file 5 — Oculoplastics outcomes reported by the hospitals. Description of the data: 11 oculoplastics outcomes reported by the hospitals. (DOCX 16 kb) [file 12886_2017_667_MOESM5_ESM.docx]

**Additional file 5** Oculoplastics outcomes reported by the hospitals

| **Institution** | **Metric** | **Hospital** | **Target** | **Reported Value** |
| --- | --- | --- | --- | --- |
| **All lid operations or unspecified** | Postoperative complications | 1/8 |  | 2.8% |
|  | Postoperative infection at 6 months | 1/8 | 0.4-1.0%^[[1]](#endnote-1)^ ^[[2]](#endnote-2)^ | 0% |
|  | Reoperation for lid surgery by 6 months | 1/8 | 2.6-8.7%^[[3]](#endnote-3)^ ^[[4]](#endnote-4)^ | 2.6% |
| **Ptosis operations** | Postoperative eyelid symmetry:  Excellent - MRD 0.5-1mm of desired  Good - MRD 1.0mm of desired | 3/8 |  | 69.7-88.5%  30.3% |
|  | Unplanned ptosis reoperations within 6 -12 months of original ptosis repair | 2/8 | <15%, 0^[[5]](#endnote-5)^ | 1.0-5% |
|  | Ptosis repair: Patient satisfaction rate  ≥ 5 on scale of 1-10  ≥ 7 on scale of 1-10 | 2/8 | 77%^[[6]](#endnote-6)^ | 94.2%  81.3% |
| **DCR** | Dacryocystorhinostomy: patient reported improved/not bothersome tearing at 6 months postoperatively | 2/8 | 80%^[[7]](#endnote-7)^ | 90% |
| **Entropion and Ectropion** | Entropion surgery success (good lid apposition) at 1 year | 2/8 | >95% | 95.397.5% |
|  | Ectropion surgery success at 1 year | 1/8 | >80% | 100% |

MRD = marginal reflection distance

1. Lee EW, Holtebeck AC, Harrison AR. Infection rates in outpatient eyelid surgery. Ophthal Plast Reconstr Surg 2009; 25(2): 109-110. [↑](#endnote-ref-1)
2. Carter SR, Stewart JM, Khan J, Archer KF, Holds JB, Seiff SR, Dailey RA. Infection after blepharoplasty with and without carbon dioxide laser resurfacing. Ophthalmology 2003; 110(7): 1430–1432. [↑](#endnote-ref-2)
3. Scoppettuolo E, Chadha V, Bunce C, Olver JM, Wright M. British Oculoplastic Surgery Society (BOPSS) National Ptosis Survey. Br J Ophthalmol 2008; 92(8): 1134–1138. 4 [↑](#endnote-ref-3)
4. Melicher J, Nerad JA. Ptosis surgery failure and reoperation. In: Cohen AJ, Weinberg DA, eds. Evaluation and management of blepharoptosis. New York: Springer; 2011: 269-274. [↑](#endnote-ref-4)
5. Scoppettuolo E, Chadha V, Bunce C, Olver JM, Wright M; BOPSS. British Oculoplastic Surgery Society (BOPSS) National Ptosis Survey. Br J Ophthalmol. 2008 Aug;92(8):1134-8. doi: 10.1136/bjo.2007.132746. Epub 2008 Jun 20. [↑](#endnote-ref-5)
6. TJ McCulley, RC Kersten, DR Kulwin, WJ Feuer. Outcome and influencing factors of external levator palpebrae superioris aponeurosis advancement for blepharoptosis. OPRS 2003;19:388–393. [↑](#endnote-ref-6)
7. Seider N, Kaplan N, Gilboa M, Gdal-On M, Miller B, Beiran I. Effect of timing of external dacryocystorhinostomy on surgical outcome. Ophthal Plast Reconstr Surg. 2007 May-Jun;23(3):183-6. [↑](#endnote-ref-7)
